# The cancer patients’ perspective on feasibility of using a fatigue diary and the benefits on self-management: results from a longitudinal study

**DOI:** 10.1007/s00520-022-07397-5

**Published:** 2022-10-13

**Authors:** Marlena Milzer, Karen Steindorf, Paul Reinke, Martina E. Schmidt

**Affiliations:** 1 Division of Physical Activity, Prevention and Cancer, German Cancer Research Center (DKFZ) and National Center for Tumor Diseases (NCT), Im Neuenheimer Feld 581, 69120 Heidelberg, Germany; 2 Medical Faculty, University of Heidelberg, Heidelberg, Germany; 3 Faculty of Medicine, University of Freiburg, Freiburg, Germany

**Keywords:** Cancer-related fatigue, Self-management, Self-monitoring, Supportive care, Symptom diary

## Abstract

**Purpose:**

To evaluate the patients’ perspective on feasibility of using a fatigue diary and its benefits on self-management.

**Methods:**

This longitudinal study enrolled 50 cancer patients in routine care. Following baseline (t0) assessment, patients were asked to complete a 7-day fatigue diary and subsequently obtained written diary evaluation. Feasibility, benefits, and fatigue-related attitudes were assessed via self-report 1 (t1) and 4 months (t2) after distributing the diary. Data were analyzed descriptively and using Wilcoxon signed-rank tests.

**Results:**

Most patients (94%) completed the diary for 7 days and rated feasibility as high. After diary completion and receiving the evaluation, fewer patients felt helpless in the face of fatigue (t1: 21% vs. t0: 53%). Additionally, more patients addressed fatigue with their general practitioner (t2: 49% vs. t0: 36%) and pro-actively searched for information and help (t2: 59% vs. t0: 38%). The diary enabled a majority of patients to be aware of their fatigue patterns, to plan daily routines accordingly and to take adequate actions against fatigue.

**Conclusion:**

The study showed that symptom monitoring via a fatigue diary was considered feasible and enhanced self-management in cancer patients. Thus, fatigue diaries might be a useful measure contributing to an improved fatigue management. The results reinforce guideline recommendations for routine application of fatigue diaries in clinical care. Healthcare professionals should encourage patients to fatigue diary use and provide individually tailored counseling based on diary entries.

**Supplementary Information:**

The online version contains supplementary material available at 10.1007/s00520-022-07397-5.

## Introduction

Numerous studies have shown that cancer-related fatigue occurs in up to 80% of the patients receiving radio- and/or chemotherapy [1] and considerably impairs quality of life [2, 3], daily life functioning, and the capacity to work [4–6]. Cancer-related fatigue has been defined by the National Comprehensive Cancer Network (NCCN) as a “distressing, persistent, subjective sense of physical, emotional and/or cognitive exhaustion related to cancer or cancer treatment that is not proportional to recent activity and interferes with usual functioning” [7].

A large body of evidence entailed the release of fatigue specific guidelines by major cancer organizations like the Canadian Association for Psychosocial Oncology (CAPO) [8], the NCCN [7], and the European Society for Medical Oncology [9] comprising recommendations for diagnostics and management of fatigue. Despite the existence of high-level evidence interventions for fatigue like physical activity and psychosocial interventions, which are concordantly recommended in the guidelines, fatigue is often disregarded and inadequately treated [7–12]. It is further proposed to provide all cancer patients with basic information on characteristics and treatment possibilities of fatigue [7]. However, there is evidence documenting a lack of information regarding fatigue in cancer patients [10]. One study revealed a deficient practice of informing about fatigue, contributing to crucial knowledge gaps in cancer patients, e.g., with regard to treatment options. Handing out a brief information booklet, however, was shown to be an effective, time- and cost-efficient intervention to enhance fatigue-related knowledge in patients [13].

Furthermore, encouraging patients to use a fatigue diary is mentioned in both the NCCN [7] and the CAPO guidelines [8]. Self-monitoring tools like symptom diaries promote self-management behaviors and positive attitudes by raising awareness of individual symptom patterns and of actions alleviating symptoms [14]. A systematic review of controlled trials, indeed, concluded that a routine assessment of patient-reported outcomes, e.g., by means of symptom diaries, could enhance symptom control, patient-clinician communication, and patient satisfaction and improve measures of supportive care [15]. Previous research on patients’ and nurses’ perspectives confirmed that for multiple symptoms arising during chemotherapy paper–pencil symptom diaries are a convenient and beneficial device, encouraging patients to discuss symptoms with healthcare professionals (HCP) and to adopt suitable self-management strategies [16]. Additionally, empirical evidence was provided for cancer patients’ acceptance and feasibility of paper–pencil symptom diaries [16]. This finding might mitigate a major concern of nurses, suspecting symptom diaries to be too burdensome to patients. Further barriers that may prevent nurses from recommending symptom diaries are perceived lack of collaboration with physicians, skepticism within the team concerning its usefulness, and practical factors like extra workload [17]. However, nurses generally acknowledge the advantages of paper–pencil diaries, i.e., enabling them to receive quick information of patients’ symptoms and to provide adequate counseling and support [17].

Further research also documented the usefulness of electronic symptom diaries and a high adherence among adolescent cancer patients showing that digital self-monitoring tools may be a relevant, convenient alternative to paper-based tools [18]. Finally, symptom diaries allow for an immediate self-report of symptoms, ensuring a more accurate overview of actual symptom severity. Since previous research found that fatigue tended to be underestimated at delayed self-report, fatigue diaries are of particular importance in order to reduce the risk of disregarding fatigue [19]. Nevertheless, feasibility and benefits of specific fatigue symptom diaries, e.g., on self-management of cancer patients have, so far, been widely neglected in previous research. Therefore, the principal goal of our study was to evaluate the feasibility and benefits of a paper–pencil-based fatigue diary for cancer patients on self-management. We further wanted to examine the patients’ perspective on several aspects of fatigue by assessing their attitudes and how they change over time after diary completion. Overall aim was to investigate whether using a fatigue diary can be one step towards an improved fatigue management.

## Methods

Our longitudinal CARPE DIEM study was conducted at the National Center for Tumor Diseases Heidelberg, Germany, between October 2019 and April 2021. Due to Covid-19, the study was on hold for 2.5 months in spring 2020. Patients were eligible for study participation if they (1) were ≥ 18 years old, (2) had a first-time diagnosis of any malignant tumor, (3) received current or completed systemic therapy or radio therapy, and (4) were able to understand and follow the study protocol. Recruitment was carried out by posters, hand-outs, and direct contact in the cancer center. The study was performed in line with the principles of the Declaration of Helsinki. Approval was granted by the Ethics Committee of the Medical Faculty of Heidelberg University. The handling of confidential information followed medical confidentiality laws, the EU General Data Protection Regulation, and the Data Protection Act of Baden-Württemberg.

At baseline (t0), fatigue symptomatology, socio-demographic data, fatigue-related attitudes, and behaviors (e.g., physical activity) were assessed via a paper–pencil questionnaire. Patients were then asked to read a brief information booklet consisting of nine easy-to-read pages and to complete the fatigue diary twice a day (at around 3 pm and 9 pm) for 7 days. Afterwards, the fatigue diary should be sent back to the study center in a prepaid envelope. Approximately 1 month (t1) and 4 months (t2) after handing out the diary patients were again asked to fill out similar questionnaires. These included the assessment of fatigue-related attitudes and items on feasibility, contents, and perceived benefits of the diary. Additionally, at t1, patients obtained a written evaluation of the diary, including a summary of their individual ratings of fatigue, sleep, and physical activity as well as evidence-based recommendations. For example, patients who were not sufficiently active were recommended to increase physical activity. They further received information and contact data of appropriate services offered in the cancer center.

### Diary

So far, German-language diaries for cancer-related fatigue did not distinguish between fatigue dimensions and only included questions on fatigue severity and activities. Therefore, the contents of our self-developed diary were based on existing diaries, but extended by recommendations of clinical guidelines [7, 9]. Thus, our fatigue diary comprised the following sections: (1) general fatigue rated on a numeric rating scale ranging from 0 = “not exhausted at all” to 10 = “completely exhausted,” and physical, emotional, and cognitive fatigue rated on a 5-point smiley face scale; (2) sleep quality and quantity, naps, and rest; (3) physical activity; and (4) exhausting and positive activities. Feasibility and understandability of the diary were pre-tested with eight patients.

### Assessments

The attitudes scale was composed of 10 statements to be rated on a 4-point Likert scale, ranging from “strongly agree” to “strongly disagree” by patients currently or recently experiencing fatigue. The statements covered, e.g., items on self-management (e.g., “I addressed my fatigue with my general practitioner (GP),” “I pro-actively searched for information, advice and help for my fatigue”) and on perceived fatigue management (e.g., “I receive a good therapy for my fatigue,” “My fatigue is not taken seriously by my treating physicians”). Apart from the attitudes scale, the t1 and t2 questionnaires comprised ratings of feasibility and perceived benefits, mainly referring to self-management behaviors, of diary completion and evaluation. For the assessment of fatigue symptomatology, the standardized EORTC QLQ-FA12 questionnaire [20] was applied. The study questionnaire was pre-tested with eight patients.

### Statistical analyses

Descriptive statistics are used to describe the sample and to present patients’ responses. Due to a non-normal distribution and an ordinally scaled dependent variable, changes in attitudes between t0 and t1 and t0 and t2, respectively, were investigated calculating Wilcoxon signed-rank tests. Since the analyses are considered exploratory, no adjustments for multiple testing were made. All statistical analyses were conducted using SAS version 9.4, with *p* ≤ 0.05 (two-tailed) considered statistically significant.

## Results

Patient characteristics are displayed in Table 1. Sixty-two patients signed informed consent and received the study package including the t0 questionnaires, the information booklet, and the diary. However, 4 patients never replied, one deceased before study completion, and 7 patients dropped out due to health issues, time constraints, or temporal stop of the study because of Covid-19 pandemic. Thus, the sample comprised 50 participants. The majority of the participants were female (84%) and the mean age of the sample was 54.3 years (SD = 13.7). Two-thirds of the participants were diagnosed with breast cancer. For 36% of the patients, the diagnosis dated back more than 12 months and 40% of the participants had metastases. Sixty-two percent of the participants were treated with chemo- and 36% with radiotherapy. Four patients had already completed cancer treatment, whereas the remaining 46 patients were on ongoing systemic or radiotherapy. Forty-four percent of the patients had a university-entrance diploma. With median EORTC QLQ-FA12 scores of 53 for physical fatigue, 33 for emotional fatigue, and 17 for cognitive fatigue, the study population expressed fatigue levels above the normative values of the general German population.

**Table 1:** Sociodemographic and medical characteristics of participants at baseline

Variable	Total (*N* = 50)	
	*M* or *n*^a^	*SD* or %
Age [years]	54.3	13.7
≤ 45 years	14	28.0%
≤ 55 years	17	34.0%
≤ 65 years	7	14.0%
≤ 75 years	8	16.0%
> 75 years	4	8.0%
Sex		
Female	42	84.0%
Male	8	16.0%
School degree		
University-entrance diploma^b^	22	44.0%
High school degree^c^	12	24.0%
Secondary school degree^d^	14	28.0%
Missing	2	4.0%
Cancer type		
Breast cancer	33	66.0%
Skin cancer	9	18.0%
Uveal cancer	2	4.0%
Other^e^	6	12.0%
Treatment		
Chemotherapy	31	62.0%
Radiotherapy	18	36.0%
Endocrine therapy	8	16.0%
Immune therapy	15	30.0%
Ongoing	46	92.0%
Completed	4	8.0%
Metastases		
No	30	60.0%
Yes	20	40.0%
Time since diagnosis		
12 + months	18	36.0%
< 12 months	13	26.0%
< 6 months	14	28.0%
Missing	5	10.0%
	*Mdn*	*Q1, Q3*
EORTC QLQ-FA12		
Physical fatigue	53	27, 80
Emotional fatigue	33	11, 44
Cognitive fatigue	17	0, 33

*M* mean, *n* number of cases, *SD* standard deviation, *Mdn* median, *Q1* first quartile, *Q3* third quartile

^a^Numbers in cells may not add up to total *N* = 50 due to missing data

^b^German “(Fach-)Abitur”

^c^German “Mittlere Reife”

^d^German “Hauptschulabschluss”

^e^Other: each *n* = 1 of lymphoma, gastric, pancreatic, lung, neuroendocrine, and endometrial cancer

Of the 50 patients included in the analyses, 48 sent their diaries back to the study center after completion. While for one participant diary entries were available for 6 days, the remaining 47 participants (94%) filled in the diary for the intended period of 7 days. Of these 47 participants, 34 completed all 126 items of the diary. The other 13 participants completed more than 90% of diary items.

Patients’ views concerning feasibility and contents of the diary are displayed in Table 2. More than 93% of the participants approved the use of smileys for rating physical, cognitive, and emotional fatigue. However, 58% found it difficult to distinguish between the three fatigue dimensions. Eighty-three percent of the participants agreed that the proposed assessment timepoints are reasonable. For a large majority of 83%, filling in the diary at 3 pm was feasible. Diary completion at 9 pm was considered feasible for 71% of the participants.

**Table 2:** Patients’ views concerning feasibility and contents of the fatigue diary

	Very much		Partly		Somewhat		Not at all	
Item	*n*^a^	%	*n*	%	*n*	%	*n*	%
Did you find the ratings of fatigue by means of smileys reasonable and appealing?	34	70.83	11	22.92	3	6.25	0	0
Did you find it difficult to rate the different types of fatigue (physical, cognitive, emotional)?	4	8.33	24	50.00	2	4.17	18	37.50
Did you find it difficult to recall sleep time and quality of the past night at 3 pm?	1	2.08	13	27.08	5	10.42	29	60.42
Did you find it reasonable to write down positive as well as exhausting activities?	21	43.75	13	27.08	14	29.17	0	0
Did you consider the proposed assessment timepoints to be reasonable?	18	37.50	22	45.83	8	16.67	0	0
Was it feasible to fill in the diary at 3 pm?	20	41.67	20	41.67	8	16.67	0	0
Was it feasible to fill in the diary at 9 pm?	15	31.25	19	39.58	10	20.83	4	8.33

*n* = number of cases

^a^Numbers in cells may not add up to total *N* = 50 due to missing data

**Fig. 1:**
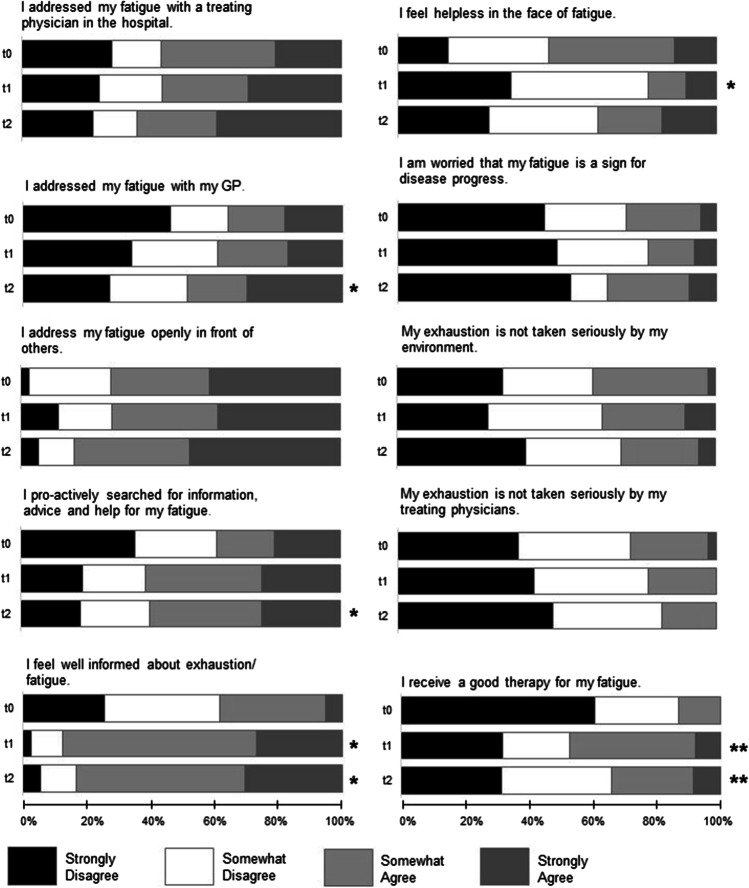
Changes in patients’ attitudes concerning fatigue between t0, t1, and t2. Statements had to be rated on a 4-point Likert scale, ranging from “strongly agree” to “strongly disagree.” t0: baseline; t1, t2: about 1 and 4 months after handing out of the diary. *p < .05; **p < .01 using Wilcoxon signed-rank test for change to baseline

Patients’ attitudes are depicted in Fig. 1. In the following, the response categories “strongly disagree”/ “somewhat disagree” and “strongly agree”/ “somewhat agree,” respectively, will be summarized. At t0, more than half of the patients (53%) strongly or somewhat agreed to feel helpless in the face of fatigue and 28% expressed to be worried that fatigue could be a sign for disease progress. Sixty-two percent of the participants did not feel well informed about fatigue and 38% stated to have searched proactively for information, advice, and help for fatigue. Concerning fatigue management, 27% agreed with the statement that their fatigue is not taken seriously by their treating physicians, and 13% affirmed to receive a good therapy for fatigue. Seventy-two percent of the participants indicated to address their exhaustion openly in front of others.

Wilcoxon signed-rank tests revealed a statistically significant difference between t0 and t1 in the item “I feel helpless in the face of fatigue” (*S* = 71.5, *p* = 0.010), with fewer people feeling helpless in the face of fatigue at t1 (21%) as opposed to t0 (53%). The comparison between t0 and t2 was not statistically significant (*p* > 0.05). Whereas no significant difference was found between t0 and t1 (*p* > 0.05), more patients addressed fatigue with their GPs at t2 (49%) compared to t0 (36%; *S* = 56, *p* = 0.030). Furthermore, significantly more patients pro-actively searched for information and support at t2 (59%) contrasted to t0 (38%; *S* = 40.5, *p* = 0.048). Significant effects were also found in the item “I feel well informed about fatigue” between t0 and both t1 (*S* = 56, *p* = 0.030) and t2 (*S* = 56, *p* = 0.030), indicating that more participants feel well informed at t1 (88%) and t2 (83%) compared to t0 (62%). Besides, significantly more participants stated to receive a good therapy for fatigue at t1 (47%, *S* = 69.5, *p* = 0.007) and t2 (34%, *S* = 54, *p* = 0.001) as opposed to t0 (13%). The other calculated Wilcoxon signed-rank tests revealed no statistically significant effects (all *p* > 0.05).

With regard to the benefits of diary completion and evaluation, two-thirds of the patients fully or partly agreed that completing the fatigue diary helped them to be more aware of their energy and fatigue levels in the course of the day (see Table 3). Moreover, diary completion and evaluation contributed to a more deliberate planning and structuring of daily routines in 80% of the participants. Sixty percent of the patients also agreed that the diary evaluation which they received by the study center was helpful for more profoundly describing their fatigue-related issues in medical consultations. The statement “The evaluation of the diary helped me taking adequate actions in respect of my fatigue” was agreed by 68% of the participants, whereas 18% agreed that it was helpful for initiating appropriate measures by HCP. For five patients, the diary was useful neither for describing their fatigue in medical consultations nor for taking adequate actions or for structuring daily routines according to energy levels. This sub-population of patients who did not benefit from the diary was female and diagnosed with non-metastatic breast cancer. Beyond that, the patients shared no characteristics like age, school degree, marital status, cancer therapy, or severity of fatigue.

**Table 3:** Perceived benefits of diary completion and evaluation^a^

	Not at all		A little		Partly		Very much	
Item	*n*^b^	%	*n*	%	*n*	%	*n*	%
The evaluation of the diary helped me to more profoundly describe my fatigue-related issues in medical consultations	4	10.53	11	28.95	18	47.37	5	13.16
The evaluation of the diary helped me with taking adequate actions in respect of my fatigue	2	4.88	11	26.83	15	36.59	13	31.71
The evaluation of the diary helped the physician/the health care professional with taking adequate actions in respect of my fatigue	11	27.50	22	55.00	6	15.00	1	2.50
Completion and evaluation of the diary contributed to a more deliberate planning and structuring of my daily routines	2	5.00	6	15.00	20	50.00	12	30.00
	Very much		Partly		Somewhat		Not at all	
	*n*	%	*n*	%	*n*	%	*n*	%
Did completion of the diary help you to be more aware of your energy and fatigue levels in the course of the day?	15	31.25	17	35.42	12	25.00	4	8.33

*n* number of cases

^a^*Diary evaluation* refers to the summary of individual ratings and evidence-based recommendations patients received by the study center after diary completion

^b^Numbers in cells may not add up to total *N* = 50 due to missing data

## Discussion

In our study, the majority of participants did not feel well informed about fatigue, was dissatisfied with fatigue treatment, and felt helpless in the face of fatigue. Additionally, more than a quarter of the participants indicated that their fatigue is not taken seriously by treating physicians. These fatigue-related attitudes at t0 reflect specific shortcomings in current fatigue management. Our results strengthen recent findings of a survey among 2508 cancer patients in which a lack of information with regard to fatigue and feelings of helplessness and of not being taken seriously by the environment were reported [10]. Previous analyses have additionally shown that poor information practices by HCPs contribute to patients’ knowledge gaps [13]. Thus, a systematic investigation of fatigue management is needed in order to identify gaps and barriers that need to be resolved for promoting guideline implementation and improving care in the field of fatigue. We are currently performing a large-scale study (LIFT project) assessing the patients’, HCPs’ and institutional perspectives on fatigue management in Germany with the aim of identifying specific starting points for improvements.

Promoting self-monitoring via a fatigue diary might be one starting point for improvement. Indeed, after completing the fatigue diary and receiving the diary evaluation by the study center, an enhanced self-management was reported by our study participants as shown in both changes in attitudes and ratings assessing the particular benefits of diary completion and evaluation. More specifically, communication with HCP appeared to be fostered by diary use since it facilitated description of symptoms and encouraged patients to address fatigue with their GPs. Improvements in self-management further relate to the findings that our patients became more aware of their energy and fatigue patterns, planned and structured their daily routines accordingly, and pro-actively searched for information. The diary also helped patients, rather than HCP, with taking adequate actions against fatigue. In turn, patients felt less helpless and more confident in coping with fatigue. After diary completion, patients further reported to receive a better therapy for their fatigue. This result, however, seems to be attributable rather to their own actions than to a better management provided by HCP, as described previously in this paragraph. Patients’ adherence to diary use was very high, indicating its feasibility.

These results support previous findings documenting beneficial effects of both paper–pencil and electronic symptom diaries for cancer patients [15, 16, 18]. In one study, using a symptom diary eased communication with HCP and improved self-management skills of cancer patients, conforming to the results of our study [16]. A recent systematic review further showed that digital self-management support tools including, among others, an information section and a symptom diary yielded positive effects on quality of life [21]. So far, just a limited number of studies has explicitly investigated the benefits of fatigue diaries. In one study, patients who kept a fatigue diary received fatigue information and regular supportive visits by nurses showed lower fatigue levels and lower distress compared to a control group [22]. Additionally, more recent findings indicate that patients’ fatigue self-efficacy can be enhanced by a digital self-management intervention consisting of educational sessions along with a fatigue diary [23]. To our knowledge, our study is the first one demonstrating the benefits of a fatigue-specific diary on self-management. Based on our findings, the use of fatigue diaries can be considered a promising measure to a better management of fatigue as it might facilitate early detection and appropriate counseling. All cancer patients should therefore be explicitly encouraged by their treating HCP to use a fatigue diary [7, 8].

But what needs to be kept in mind when recommending fatigue diary use? First of all, using a fatigue diary should always be accompanied by sufficient information about characteristics, possible causes, and treatment options of fatigue. This is consistent with a publication suggesting that generally, self-management programs should comprise information as well as tools for enhancing perception of control and self-efficacy [24]. Furthermore, similarly to our diary, assessments of sleep, physical activity, and positive and exhausting activities should be included as they are required for adequate counseling and treatment by HCP. Although half of our participants had problems discriminating between the three fatigue dimensions, it might still be relevant to measure them separately and to not only use a general fatigue rating scale. This can be justified by previous research showing partly different determinants of physical, emotional, and cognitive fatigue possibly entailing different treatments [25]. Most importantly, to maintain patients’ motivation for diary use, it is crucial to not only recommend a fatigue diary or hand it out, but in a next step to also incorporate patients’ diary entries in appointments and to provide individually tailored support [16]. In our study, this aspect was ensured by the diary evaluations participants received by the study center. These consisted of a summary of their ratings, individual recommendations, and contact information for further support.

Previous research showed that HCP, more specifically, nurses generally acknowledge usefulness of symptom diaries and argue that integration of such diaries into daily practice is feasible [17]. However, implementation of symptom diaries in oncology is impeded by different barriers, e.g., HCPs’ concern of patients perceiving diaries as too burdensome [17]. Feedback of our study participants did not support these concerns. In contrast, a large majority of the patients judged the fatigue diary as helpful and feasible, strengthening evidence concerning patients’ views on feasibility of symptom diaries in cancer care [16]. Given the feasibility and benefits of using a fatigue diary, efforts should be made in order to accelerate its implementation. Previous research has already named several factors that need to be taken into account in order to promote the implementation of symptom diaries in oncology, e.g., trainings for HCP, the involvement of interdisciplinary teams, and monitoring of the implementation process by means of the plan-do-check-act cycle, a scientific method applied in many areas for quality improvement [26]. But firstly, raising awareness for the relevance of self-monitoring tools for fatigue in both HCP and patients is essential. However, implementation of fatigue diaries in clinical practice might be a long, challenging process. Important questions like “who should be responsible for discussing fatigue diaries with patients?” and “what is required to integrate diaries in existing workflows?” still need to be figured out. Our ongoing LIFT project will provide answers to these questions and point out specific starting points for implementation.

Some limitations of the study need to be considered. First, a selection bias cannot be excluded as only patients willing to complete the diary and to read the information booklet consented to participate. Furthermore, the sample was made up of only 50 patients, mainly diagnosed with breast cancer, preventing a generalizability of the results. Thus, future research should include a larger sample, encompassing different cancer types. Although the longitudinal design of the study can be regarded as a major strength, the study is limited by its exploratory design. For this reason, randomized controlled trials testing the specific benefits of fatigue diaries, e.g., on self-management, should be conducted. Besides, due to our research approach, it cannot be concluded whether changes in attitudes are attributed specifically to the diary, to the information booklet, to the combination of both, or to other factors. However, as mentioned above, it can be objected that using a fatigue diary should always be preceded by information and education and, thus, cannot be considered independently. Since no validated instrument for the assessment of self-management was used, it can be argued whether our self-developed items actually measured self-management.

Additionally, digital tools including symptom self-reports have become increasingly important in cancer care due to feasibility and multiple clinical benefits [21, 27, 28]. Therefore, assessing the effects of digital fatigue-specific diaries is an important issue to be addressed in the future. However, due to a certain number of people without access to appropriate electronic devices or with insufficient skills to use digital tools, presumably particularly among the elderly patients, paper–pencil diaries stay an important alternative [17].

## Conclusion

This study provides valuable insights into cancer patients’ attitudes towards fatigue. Baseline attitudes such as self-reported knowledge gaps concerning fatigue and feelings of helplessness and of not being taken seriously by HCP reflect shortcomings in current fatigue management. Hence, efforts should be made in order to improve the situation for cancer patients affected by fatigue. Self-monitoring by means of a fatigue diary might be one useful and easily applicable measure contributing to the improvement of fatigue care. Indeed, this study showed that using a paper–pencil-based 7-day diary for fatigue enhanced self-management in patients diagnosed with cancer. Using a fatigue diary was further found to be feasible to patients. These findings reinforce recommendations of the NCCN and CAPO for routine application of fatigue diaries in clinical care. HCP should therefore encourage cancer patients to use a fatigue diary and may provide individually tailored counseling based on patients’ diary entries. However, HCP might be faced with barriers hampering implementation into clinical practice. It is therefore crucial to consider the HCPs’ perspective on fatigue diaries in future research to identify the steps which are required for successful implementation. Furthermore, it should be investigated how the findings of this study can be applied to digital tools.

## Supplementary Information

Below is the link to the electronic supplementary material.

Supplementary file1 (PDF 218 KB)

Supplementary file2 (PDF 164 KB)

Supplementary file3 (PDF 114 KB)

## Data Availability

Data can be made available to scientific cooperation partners on request.

## Code availability

N/A.

## References

[1] Henry DH, Viswanathan HN, Elkin EP, Traina S, Wade S, Cella D (2008) Symptoms and treatment burden associated with cancer treatment: results from a cross-sectional national survey in the U.S. Support Care Cancer 16(7):791–801. 10.1007/s00520-007-0380-2 10.1007/s00520-007-0380-2 18204940

[2] Minton O, Berger A, Barsevick A, Cramp F, Goedendorp M, Mitchell SA, Stone PC (2013). Cancer-related fatigue and its impact on functioning. Cancer.

[3] Schmidt ME, Chang-Claude J, Vrieling A, Heinz J, Flesch-Janys D, Steindorf K (2012). Fatigue and quality of life in breast cancer survivors: temporal courses and long-term pattern. J Cancer Surviv.

[4] Roila F, Fumi G, Ruggeri B, Antonuzzo A, Ripamonti C, Fatigoni S, Cavanna L, Gori S, Fabi A, Marzano N, Graiff C, De Sanctis V, Mirabile A, Serpentini S, Bocci C, Pino MS, Cilenti G, Verusio C, Ballatori E, Network Italiano per le Cure di Supporto in Oncologia (2019) Prevalence, characteristics, and treatment of fatigue in oncological cancer patients in Italy: a cross-sectional study of the Italian Network for Supportive Care in Cancer (NICSO). Support Care Cancer 27(3):1041-104710.1007/s00520-018-4393-9 10.1007/s00520-018-4393-9 30084104

[5] Jones JM, Olson K, Catton P, Catton CN, Fleshner NE, Krzyzanowska MK, McCready DR, Wong RK, Jiang H, Howell D (2016). Cancer-related fatigue and associated disability in post-treatment cancer survivors. J Cancer Surviv.

[6] Schmidt ME, Scherer S, Wiskemann SK (2019). Return to work after breast cancer: the role of treatment-related side effects and potential impact on quality of life. Eur J Cancer Care.

[7] National Comprehensive Cancer Network (NCCN) (2022) NCCN Clinical Practice Guidelines in Oncology: Cancer-related fatigue (Version 2.2022). Retrieved February 10, 2022 from: https://www.nccn.org/login?ReturnURL=https://www.nccn.org/professionals/physician_gls/pdf/fatigue.pdf.

[8] Howell D, Keshavarz H, Broadfield L, Hack T, Hamel M, Harth T, Jones J, McLeod D, Olson K, Phan S, Sawka A, Swinton N, Ali M; on behalf of the Cancer Journey Advisory Group of the Canadian Partnership Against Cancer (2015) A pan Canadian practice guideline for screening, assessment, and management of cancer-related fatigue in adults version 2. Canadian Partnership Against Cancer (Cancer Journey Advisory Group) and the Canadian Association of Psychosocial Oncology, Toronto. Available on http://www.capo.ca

[9] Fabi A, Bhargava R, Fatigoni S, Guglielmo M, Horneber M, Roila F, Weis J, Jordan K, Ripamonti CI, Guidelines Committee ESMO (2020). Cancer-related fatigue: ESMO Clinical Practice Guidelines for diagnosis and treatment. Ann Oncol.

[10] Schmidt ME, Bergbold S, Hermann S, Steindorf K (2021). Knowledge, perceptions, and management of cancer-related fatigue: the patients' perspective. Support Care Cancer.

[11] Haussmann A, Schmidt ME, Illmann ML, Schröter M, Hielscher T, Cramer H, Maatouk I, Horneber M, Steindorf K (2022) Meta-analysis of randomized controlled trials on yoga, psychosocial, and mindfulness-based interventions for cancer-related fatigue: what intervention characteristics are related to higher efficacy? Cancers 14(8). 10.3390/cancers14082016 10.3390/cancers14082016 PMC9032769 35454922

[12] Tomlinson D, Diorio C, Beyene J, Sung L (2014). Effect of exercise on cancer-related fatigue: a meta-analysis. Am J Phys Med Rehabil.

[13] Schmidt ME, Milzer M, Weiß C, Reinke P, Grapp M, Steindorf K (2022). Cancer-related fatigue: benefits of information booklets to improve patients' knowledge and empowerment. Support Care Cancer.

[14] Richard A, Shea K (2011). Delineation of self-care and associated concepts. J Nurs Scholarsh.

[15] Kotronoulas G, Kearney N, Maguire R, Harrow A, Di Domenico D, Croy S, MacGillivray S (2014). What is the value of the routine use of patient-reported outcome measures toward improvement of patient outcomes, processes of care, and health service outcomes in cancer care? A systematic review of controlled trials. J Clin Oncol.

[16] Coolbrandt A, Steffens E, Wildiers H, Bruyninckx E, Verslype C, Milisen K (2017). Use of a symptom diary during chemotherapy: a mixed-methods evaluation of the patient perspective. Eur J Oncol Nurs.

[17] Coolbrandt A, Bruyninckx E, Verslype C, Steffens E, Vanhove E, Wildiers H, Milisen K (2017). Implementation and use of a patient symptom diary during chemotherapy: a mixed-methods evaluation of the nurse perspective. Oncol Nurs Forum.

[18] Baggott C, Gibson F, Coll B, Kletter R, Zeltzer P, Miaskowski C (2012). Initial evaluation of an electronic symptom diary for adolescents with cancer. JMIR Res Protoc.

[19] Coolbrandt A, Van den Heede K, Vanhove E, De Bom A, Milisen K, Wildiers H (2011). Immediate versus delayed self-reporting of symptoms and side effects during chemotherapy: does timing matter?. Eur J Oncol Nurs.

[20] Weis J, Tomaszewski KA, Hammerlid E, Ignacio Arraras J, Conroy T, Lanceley A, Schmidt H, Wirtz M, Singer S, Pinto M, Alm El-Din M, Compter I, Holzner B, Hofmeister D, Chie WC, Czeladzki M, Harle A, Jones L, Ritter S, Flechtner HH, Bottomley A, EORTC Quality of Life Group (2017) International psychometric validation of an EORTC quality of life module measuring cancer related fatigue (EORTC QLQ-FA12). J Natl Cancer I 109(5):djw273. 10.1093/jnci/djw273 10.1093/jnci/djw273 28376231

[21] Adriaans DJ, Dierick-van Daele AT, van Bakel MJHM, Nieuwenhuijzen GA, Teijink JA, Heesakkers FF, van Laarhoven HW (2021). Digital self-management support tools in the care plan of patients with cancer: review of randomized controlled trials. J Med Internet Res.

[22] Ream E, Richardson A, Alexander-Dann C (2006). Supportive intervention for fatigue in patients undergoing chemotherapy: a randomized controlled trial. J Pain Symptom Manage.

[23] Foster C, Grimmett C, May CM, Ewings S, Myall M, Hulme C, Smith PW, Powers C, Calman L, Armes J, Breckons M, Corner J, Fenlon D, Batehup L, Lennan E, May C, Morris C, Neylon A, Ream E, Turner L, Yardley L, Richardson A (2016). A web-based intervention (RESTORE) to support self-management of cancer-related fatigue following primary cancer treatment: a multi-centre proof of concept randomised controlled trial. Support Care Cancer.

[24] Kidd L, Hubbard G, O’Carroll R, Kearney N (2009). Perceived control and involvement in self care in patients with colorectal cancer. J Clin Nurs.

[25] Schmidt ME, Wiskemann J, Schneeweiss A, Potthoff K, Ulrich CM, Steindorf K (2018). Determinants of physical, affective, and cognitive fatigue during breast cancer therapy and 12 months follow-up. Int J Cancer.

[26] IJzerman-Korevaar M, de Graeff A, Heijckmann S, Zweers D, Vos BH, Hirdes M, Witteveen PO, Teunissen SCCM (2021) Use of a symptom diary on oncology wards: effect on symptom management and recommendations for implementation. Cancer Nurs 44(4):E209-E220. 10.1097/NCC.0000000000000792 10.1097/NCC.0000000000000792 31990694

[27] LeBlanc TW, Abernethy AP (2017). Patient-reported outcomes in cancer care—hearing the patient voice at greater volume. Nat Rev Clin Oncol.

[28] Basch E, Deal AM, Kris MG, Scher HI, Hudis CA, Sabbatini P, Rogak L, Bennett AV, Dueck AC, Atkinson TM, Chou JF, Dulko D, Sit L, Barz A, Novotny P, Fruscione M, Sloan JA, Schrag D (2016). Symptom monitoring with patient-reported outcomes during routine cancer treatment: a randomized controlled trial. J Clin Oncol.

