# Supplementary material for: The cancer patients’ perspective on feasibility of using a fatigue diary and the benefits on self-management: results from a longitudinal study
Source: Support Care Cancer. 2022 Oct 13;30(12):10213–21. doi: 10.1007/s00520-022-07397-5 (PMC9559147; doi:10.1007/s00520-022-07397-5)
Supplement: Supplementary file 1 — Supplementary file1 (PDF 218 KB) [file 520_2022_7397_MOESM1_ESM.pdf]

Supplementary material: Excerpts from an exemplary diary evaluation (translated from German)

- *After sending the completed diary back to the study center patients received written evaluation of the diary, including individually-tailored, evidence-based recommendations*

1. **Conclusion**

In summary, your diary entries indicate that you suffer from mild to moderate exhaustion symptoms. In the course of the day your exhaustion increased continuously. Positive and supporting activities were the times you spent outdoors as well as activities with friends or family. It can be presumed that strong exhaustion symptoms were caused by nocturnal muscle cramps and headache resulting in a poor sleep quality. Generally, you reported a moderate to good sleep quality. Your sleep duration was in the normal range. Further positive activities you mentioned within the diary comprised reading and listening to music.

Recommendations

The results of your diary evaluation show that you occasionally suffer from **physical exhaustion**. The approaches listed in the information brochure could be useful for you. If the exhaustion is worsening or if you feel burdened by the exhaustion, we recommend reaching out to your physician in order to discuss these problems.

It is very positive, that you are already physically active. However, if you would like to further enhance your fitness and muscle strength, which could contribute to a reduction of your exhaustion, you can contact the NCT service specialized for physical activity and exercise.

Contact data of the service [...]

Furthermore, your diary evaluation shows that you sometimes suffer from **emotional and cognitive exhaustion**. You can find more information and suitable treatment options in the information brochure. Additionally, you and your relatives can contact the psycho-oncological service of the cancer center in order to receive support for coping with fatigue and with the cancer disease.

Contact data of the service [...]

In case of questions you may also contact our study center.

Contact data of the study center [...]

2. **Possible correlation between exhaustion and your mean physical activity level**

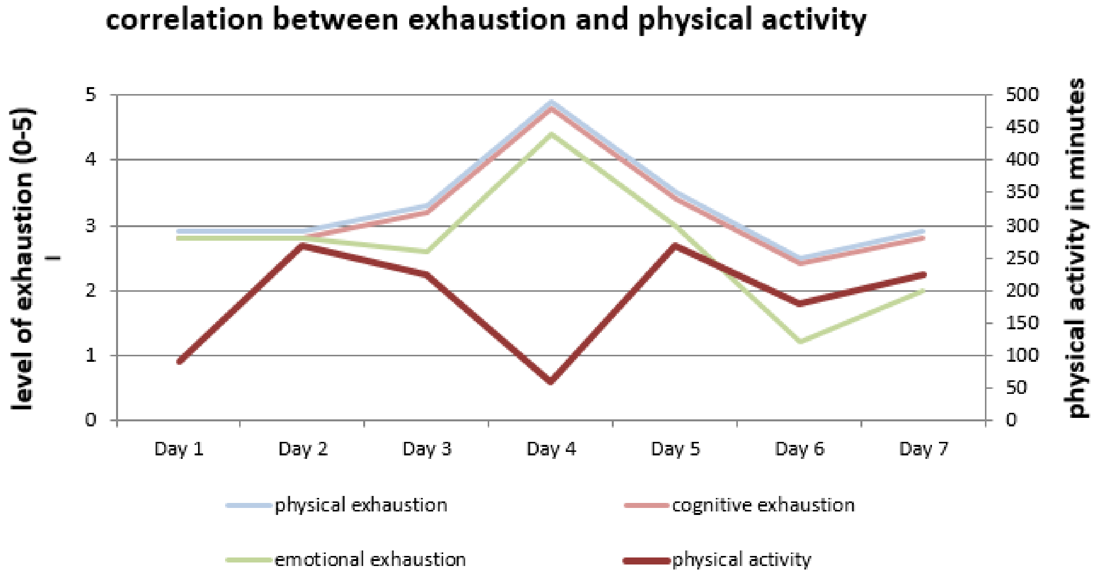

The red line represents your physical activity. It can be seen that a higher general exhaustion was not associated with higher levels of physical activity. Especially in the beginning of the week you felt tired despite low levels of physical activity. When your exhaustion increased on the fourth day, your physical activity was reduced. Your physical exhaustion and cognitive exhaustion levels were comparable. Despite high levels of exhaustion, you were physically active for more than 150 minutes for five days, which is very good. There is evidence that physical activity has positive effects on well-being and self-confidence. Furthermore, you should maintain meetings with colleagues, friends and relatives as they made you feel better.

3. **Your exhaustion in the course of the day and the week**

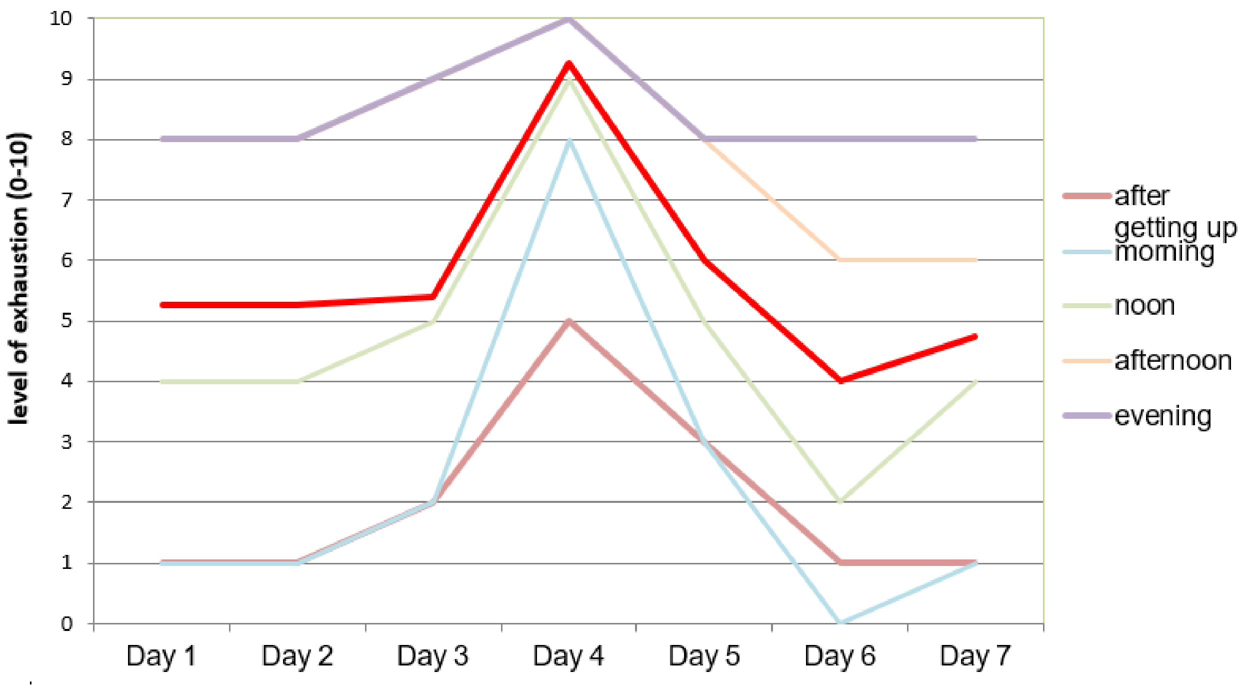

3.1 Daily and weekly overview of the different types of exhaustion

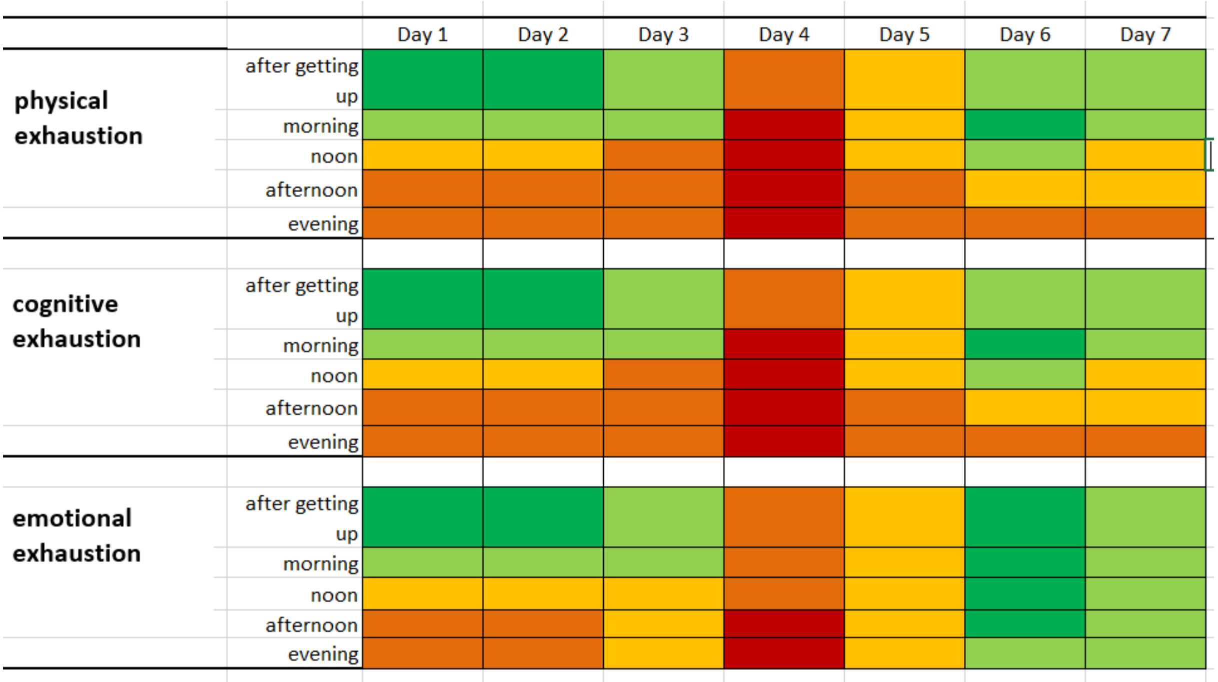

<u>In the course of the week</u>

Your exhaustion was highest in the beginning and in the middle of the diary week. Especially your emotional tiredness decreased in the end of the week. Generally, your physical and cognitive exhaustion were more pronounced than emotional fatigue.

<u>In the course of the day</u>

Your exhaustion generally increased in the course of the day. The diary entries indicated that the fourth day was the worst one, possibly attributed to nocturnal muscular tensions and headache.

4. **Your sleep**

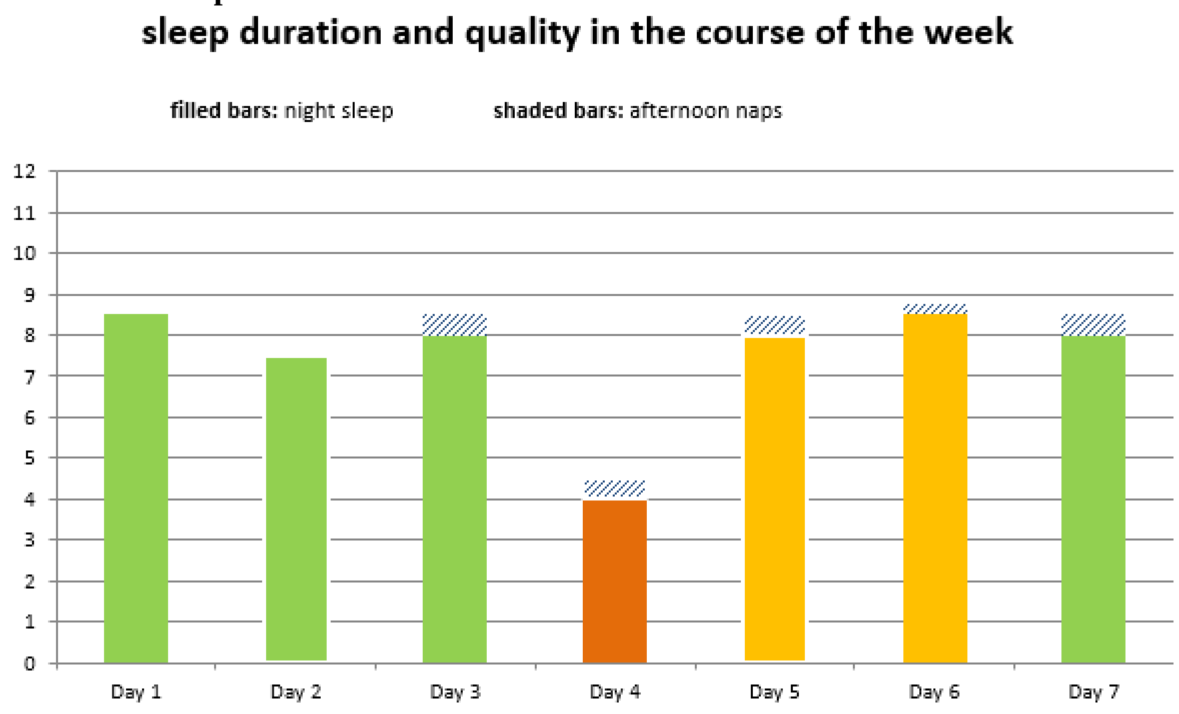

Sleep duration: According to the recommendations published by the National Health Foundation (7-9 hours), your sleep duration is in the normal range. You went to bed at approximately the same time every day.

Sleep quality: You rated your sleep quality as moderate. According to your diary entries it can be presumed that outdoors physical activity as well as spending time with friends, relatives etc. positively influenced your sleep quality.

5. **Your physical activity**

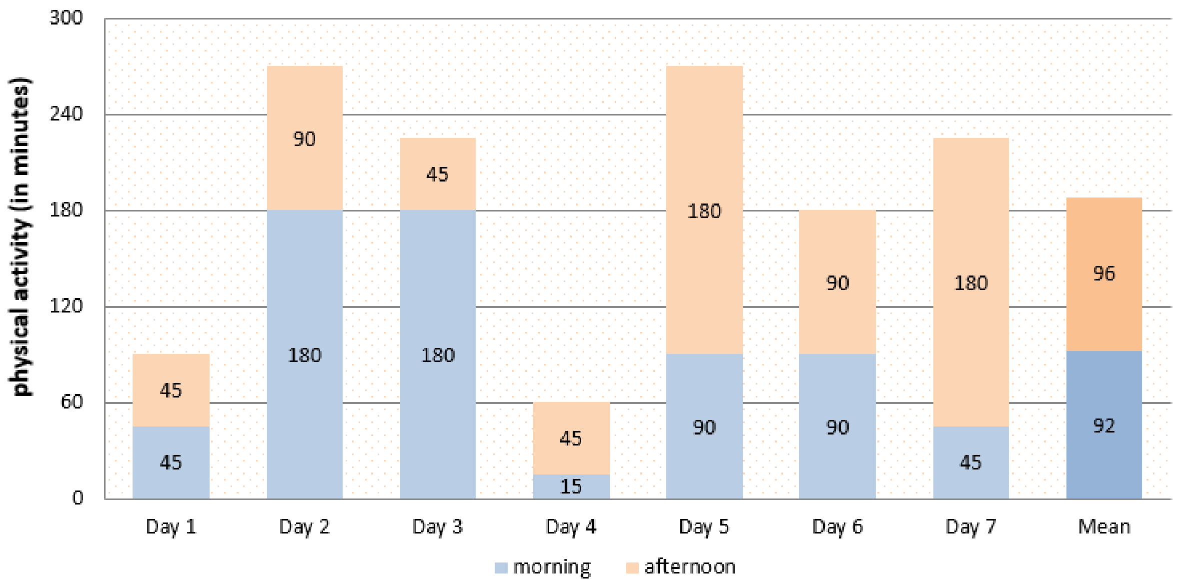

Physical activity: Blue areas of the diagram represent the amount of time (in minutes) you spent physically active in the morning whereas the beige areas show your physical activity in the afternoon. You tended to be more physically active in the afternoon. The World Health Organization recommends either 150 minutes of moderate physical activity or 75 minutes of vigorous physical activity per week. They further advise resistance exercises twice per week.

As shown in the diagram you complied with the recommendations. Regular group activities or family activities like walks or sports programs might help to further increase physical activity. If, so far, you have not yet integrated resistance training in your everyday life, this might be a good addition – given that your skills and health status allow for it.
