# Supplementary material for: The cancer patients’ perspective on feasibility of using a fatigue diary and the benefits on self-management: results from a longitudinal study
Source: Support Care Cancer. 2022 Oct 13;30(12):10213–21. doi: 10.1007/s00520-022-07397-5 (PMC9559147; doi:10.1007/s00520-022-07397-5)
Supplement: Supplementary file 2 — Supplementary file2 (PDF 164 KB) [file 520_2022_7397_MOESM2_ESM.pdf]

Supplementary material: Excerpts from the fatigue diary (translated from German)

- *The questions were equivalently asked in the evening ratings at approximately 9pm*
- *The diary comprised ratings for seven consecutive days*

DAY 1

Please fill in today's weekday: ____

Ratings at 3pm

When did you go to bed last night? at _ _ : _ _ am/ pm

When did you wake up this morning? at _ _ : _ _ am

When did you get up today? at _ _ : _ _ am

Sleep quality: ☐ ☐ ☐ ☐ ☐

If you indicated a poor sleep quality you may write down possible reasons like rumination, noise, hot flashes etc. in the following lines:

____

____

____

Please indicate how exhausted you felt **about 30 minutes after getting up:**

not exhausted at all ____ completely exhausted

☐	☐	☐	☐	☐	☐	☐	☐	☐	☐	☐
0	1	2	3	4	5	6	7	8	9	10

Type of exhaustion [green = not exhausted at all, red = completely exhausted)

Physical exhaustion: ☐ ☐ ☐ ☐ ☐ e.g. powerless, drowsy, every moment is arduous…

Cognitive exhaustion: ☐ ☐ ☐ ☐ ☐ e.g. forgetful, lacking in concentration…

Emotional exhaustion: ☐ ☐ ☐ ☐ ☐ e.g. down, discouraged, listless…

Please indicate how exhausted you felt **<u>in the morning (getting up until noon)</u>**

not exhausted at all										completely exhausted
☐	☐	☐	☐	☐	☐	☐	☐	☐	☐	☐
0	1	2	3	4	5	6	7	8	9	10

Type of exhaustion [green = not exhausted at all, red = completely exhausted)

	green				red	
Physical exhaustion:	☐	☐	☐	☐	☐	e.g. powerless, drowsy, every moment is arduous…
Cognitive exhaustion:	☐	☐	☐	☐	☐	e.g. forgetful, lacking in concentration…
Emotional exhaustion:	☐	☐	☐	☐	☐	e.g. down, discouraged, listless…

Please indicate how exhausted you felt **<u>in the early afternoon (noon until 3pm)</u>**

not exhausted at all										completely exhausted
☐	☐	☐	☐	☐	☐	☐	☐	☐	☐	☐
0	1	2	3	4	5	6	7	8	9	10

Type of exhaustion [green = not exhausted at all, red = completely exhausted)

	green				red	
Physical exhaustion:	☐	☐	☐	☐	☐	e.g. powerless, drowsy, every moment is arduous…
Cognitive exhaustion:	☐	☐	☐	☐	☐	e.g. forgetful, lacking in concentration…

Emotional exhaustion:	☐	☐	☐	☐	☐	e.g. down, discouraged, listless…

So far, how much time did you spend **physically active** today (e.g. walking, cycling, domestic work…)?

☐ less than 30 minutes
☐ more than 30 minutes, but less than an hour
☐ more than an hour, but less than two hours
☐ more than two hours, but less than four hours
☐ four hours or more

Between getting up and 3pm, did you lie down for a while and/or have a nap?

☐ no
☐ yes → between _ _ : _ _ am/pm and _ _ : _ _ am/pm

So far, what was particularly exhausting today (e.g. walking to the bakery, concerns regarding the medical checkup etc.)?

So far, what were positive activities that made you feel better today (e.g. listening to music, meeting the neighbor etc.)?
