# Supplementary material for: The cancer patients’ perspective on feasibility of using a fatigue diary and the benefits on self-management: results from a longitudinal study
Source: Support Care Cancer. 2022 Oct 13;30(12):10213–21. doi: 10.1007/s00520-022-07397-5 (PMC9559147; doi:10.1007/s00520-022-07397-5)
Supplement: Supplementary file 3 — Supplementary file3 (PDF 114 KB) [file 520_2022_7397_MOESM3_ESM.pdf]

Supplementary material: Excerpts from our study questionnaires including the aspects addressed in the manuscript (translated from German)

- *t0 = baseline; t1 = about one month after reading the information booklet; t2 = about four months after reading the information booklet*
- *numbering of the questions doesn't correspond to the original questionnaires*

1. **Patients sometimes report that they have the following symptoms or problems. Please indicate the extent to which you have experienced these symptoms or problems <u>during the past week</u>. Please answer by ticking the option that best applies to you.** *[question was asked at t0 only; EORTC QLQ-FA12]*

	**During the past week:**	not at all	a little	quite a bit	very much
1.	Have you lacked energy?	☐	☐	☐	☐
2.	Have you felt exhausted?	☐	☐	☐	☐
3.	Have you felt slowed down?	☐	☐	☐	☐
4.	Did you feel sleepy during the day?	☐	☐	☐	☐
5.	Did you have trouble getting things started?	☐	☐	☐	☐
6.	Did you feel discouraged?	☐	☐	☐	☐
7.	Did you feel helpless?	☐	☐	☐	☐
8.	Did you feel frustrated?	☐	☐	☐	☐
9.	Did you have trouble thinking clearly?	☐	☐	☐	☐
10.	Did you feel confused?	☐	☐	☐	☐
11.	Did tiredness interfere with your daily activities?	☐	☐	☐	☐
12.	Did you feel that your tiredness is (was) not understood by the people who are close to you ?	☐	☐	☐	☐

2. **To what extent to the following statements apply to you?** *[question was asked at t0 only; PSEFSM]*

Please tick the most appropriate option on the scale ranging from "very uncertain" to "very certain".

	very uncertain										very certain
How certain are you that you can control your fatigue?	☐	☐	☐	☐	☐	☐	☐	☐	☐	☐	☐
How certain are you that you can regulate your activity so as to be active without aggravating your fatigue?	☐	☐	☐	☐	☐	☐	☐	☐	☐	☐	☐
How certain are you that you can do something to help yourself feel better if you are feeling fatigued?	☐	☐	☐	☐	☐	☐	☐	☐	☐	☐	☐
As compared with other people with fatigue like yours, how certain are you that you can manage fatigue during your daily activities?	☐	☐	☐	☐	☐	☐	☐	☐	☐	☐	☐
How certain are you that you can manage your fatigue symptoms so that you can do the things you enjoy doing?	☐	☐	☐	☐	☐	☐	☐	☐	☐	☐	☐
How certain are you that you can deal with the frustration of fatigue?	☐	☐	☐	☐	☐	☐	☐	☐	☐	☐	☐

3. **Do you feel extremely exhausted since cancer diagnosis/ treatment?** *[question was asked at t0, t1 and t2]*
   - ☐ never/rarely
   - ☐ sometimes
   - ☐ often
   - ☐ always

   (sometimes / often / always) → *please continue with question 1.1*

3.1 To what extent do the following statements apply to you personally? *[question was asked at t0, t1 and t2]*

		fully disagree	rather disagree	rather agree	fully agree
1.	My exhaustion is not taken seriously by my environment.	☐	☐	☐	☐

2.	My exhaustion is not taken seriously by my treating physicians.	☐	☐	☐	☐
3.	I address my fatigue openly in front of others.	☐	☐	☐	☐
4.	I feel helpless in the face of fatigue.	☐	☐	☐	☐
5.	I am worried that my fatigue is a sign for disease progress.	☐	☐	☐	☐
6.	I addressed my fatigue with a treating physician in the hospital.	☐	☐	☐	☐
7.	I addressed my fatigue with my GP.	☐	☐	☐	☐
8.	I pro-actively searched for information, advice and help for my fatigue.	☐	☐	☐	☐
9.	I feel well informed about exhaustion/ fatigue.	☐	☐	☐	☐
10.	I receive a good therapy for my fatigue.	☐	☐	☐	☐

4. Questions regarding content and layout of the diary *[question was asked at t1 only]*
For the following questions please tick the answer that best applies to you.

	very much	partly	somewhat	not at all
Did you find the evaluation of fatigue by means of smileys reasonable and appealing?	☐	☐	☐	☐
Did you find it difficult to rate the different types of fatigue (physical, cognitive, emotional)?	☐	☐	☐	☐
Did you find it difficult to recall sleep time and quality of the past night at 3pm?	☐	☐	☐	☐
Did completion of the diary help you to be more aware of your energy and fatigue levels in the course of the day?	☐	☐	☐	☐
Did you find it reasonable to note positive as well as exhausting activities?	☐	☐	☐	☐

Did you consider the proposed assessment timepoints to be reasonable?	☐	☐	☐	☐
Was it feasible to fill in the diary at 3pm?	☐	☐	☐	☐
Was it feasible to fill in the diary at 9pm?	☐	☐	☐	☐

If filling in the diary at the proposed assessment timepoints was (partly) difficult for you, which timepoints would you have preferred?

timepoint in the afternoon _ _ : _ _ , timepoint in the evening _ _ : _ _

Further comments or suggestions concerning the diary: *[t1]*

5. To what extent was the diary beneficial to you? *[question was asked at t2 only]*

For the following statements please tick the answer that best applies to you.

	fully disagree	rather disagree	rather agree	fully agree
The evaluation of the diary was helpful for more profoundly describing my fatigue-related issues in medical consultations.	☐	☐	☐	☐
The evaluation of the diary helped me with taking adequate actions in respect of my fatigue.	☐	☐	☐	☐
The evaluation of the diary helped the physician/ the healthcare professional with taking adequate actions in respect of my fatigue.	☐	☐	☐	☐
Completion and evaluation of the diary contributed to a more deliberate planning and structuring of my daily routines.	☐	☐	☐	☐
